# Knowledge, attitude and practice towards voluntary counseling and testing among university students in North West Ethiopia: a cross sectional study

**DOI:** 10.1186/1471-2458-13-714

**Published:** 2013-08-02

**Authors:** Zelalem Addis, Aregawi Yalew, Yitayal Shiferaw, Abebe Alemu, Wubet Birhan, Biniam Mathewose, Belayenesh Tachebele

**Affiliations:** 1 University of Gondar, College of Medicine and Health Science, School of Biomedical and Laboratory Sciences, P.O. Box 196, Gondar, Ethiopia; 2 University of Gondar, Hospital Laboratory, P.O. Box 196, Gondar, Ethiopia

**Keywords:** Voluntary counseling and testing, Knowledge, Attitude, Practice

## Abstract

**Background:**

Voluntary counseling and testing (VCT) is one among different approaches which have been implemented as an attempt to slow the spread of HIV infection and minimize its impact at the individual, family and society level. VCT is perceived to be an effective strategy in risk reduction among sexually active young people like tertiary level students. Ethiopia as a country with high burden of HIV started responding to the epidemic by preparing and updating guidelines on VCT. The objective of this study was to assess the level of knowledge, attitude and practice of Voluntary Counseling and Testing (VCT) for HIV among university students in North West Ethiopia.

**Methods:**

A cross sectional study was conducted from February to May 2010 using a stratified sampling method to enroll students from different faculties into the study. A total of 330 university students filled in a self-administered questionnaire with response rate of 97.3%. Main outcome measures included level of knowledge, attitude and practice of VCT for HIV. A chi-square test was used to determine an association between a number of independent factors and dependant variables.

**Result:**

About 66.1% of the study participants were males with a mean age of 20 years. Majority (75.6%) of the respondents were Orthodox with 63% reported living in urban areas before joining the university. From the study participants 86.3% were knowledgeable on VCT, 73.3% had positive attitude towards VCT for HIV and 61.8% had had VCT for HIV in the past. Previous residence before joining the university, level of education, sex and religion were among the sociodemographic variables that showed statistically significant association with the one or more of the outcome variables. Fear of positive results, stigma and discrimination following the positive results were reported as main barriers for VCT uptake.

**Conclusion:**

The findings reveal important barriers for VCT uptake and suggest strategies to reduce stigma and discrimination.

## Background

Acquired immunodeficiency syndrome (AIDS) caused by the human immunodeficiency virus (HIV) is a major health problem in many parts of the world, and is considered as a pandemic disease [1]. By the year 2010, the World Health Organization (WHO) estimated 34 million people living with HIV and an estimated 1.8 million deaths around the world [2]. Sub-Saharan Africa remains the region most heavily affected by HIV. In 2010, about 68% of all people living with HIV resided in sub-Saharan Africa. Sub-Saharan Africa also accounted for 70% of new HIV infections and almost half of the deaths from AIDS related illness in 2010 [3]. Almost a quarter of people living with HIV are under the age of 25. Young people ages 15–24 represent 45% of all new HIV infections. In sub-Saharan Africa, nearly 3.3 million youth are living with HIV. Lack of information, skills, and access to services for youth is expected to fuel the epidemic [4].

Ethiopia, as a country in the Sub-Saharan region, is a country with high HIV prevalence. According to the single point estimate, the Ethiopian adult HIV prevalence was 2.2% in 2008 with an estimated 1,037,267 people living with HIV in the country [5]. According to the Ethiopian demographic and health survey report of 2011, the percentage of HIV positive in the age group 15–24 years was less than one percent [6].

Many countries have been trying to take many different approaches in an attempt to slow the spread of HIV infection and minimize its impact on the individual, family and society. Among these strategies include; voluntary counseling and testing (VCT), provider initiated counseling and testing (PICT), diagnosis of HIV in infants and young children, family care and partner testing and counseling based on index care, condom promotion and provision, detection and management of sexually transmitted infections, safer sex and risk reduction counseling, male circumcision, targeted interventions for sex workers and homosexuals [7]. Among these VCT is internationally recognized as an effective and important strategy for both prevention and care of HIV [8].

VCT is the process by which an individual undergoes counseling enabling him or her to make an informed choice about being tested for HIV. This decision must be entirely the choice of the individual and he or she must be assured that the process will be confidential [9].

VCT is an effective strategy for facilitating behavioral change around both preventing HIV as well as getting early access to care and support. It is also instrumental in bringing about behavioral change, reducing unprotected sex and helping reduce the incidence of HIV and other STIs [10]. However, the availability of VCT services in Ethiopia has been uneven, and even when available, uptake has been relatively low [11].

Ethiopia responded to the HIV epidemic as early as 1985 by developing polices different guidelines (PMTCT, ART, IP, VCT etc.) and strategic documents to create an environment conducive for the implementation of HIV prevention, care, and treatment and support programs. As part of this effort, the first counselling and testing guidelines were published by the Federal Ministry of Health (FMOH) in 1996 and subsequently edited in 2002 and 2007 [11].

Studies in different areas indicated that knowledge, attitude and practice of tertiary school students towards VCT is low and its uptake is minimal. The low uptake was found to be associated with ignorance, fear of being positive, cost of VCT, inadequate number of VCT centers and stigmatization constituted major hindrances to acceptances of VCT for HIV [7,8,12-14].

There are reports on the awareness and uptake of VCT service among different study groups in the study area [10,15]. However, there are no studies conducted on the knowledge, attitude and practice of University students towards VCT service. Interventions developed for general population may not be appropriate for university students. In order to develop the body of knowledge needed to develop interventions targeted to different category of people, it is necessary to study the overall KAP of university students towards VCT in a variety of institutional settings. The university environment offers great opportunity for HIV high-risk behaviors, including unsafe sex [16]. University students are at risk because they tend to be sexually adventurous, often with multiple partners and do not consistently use condoms [17,18]. Moreover youths, students of tertiary level, constitute a significant proportion of persons affected by HIV and a good number of them are also sexually active. Knowing the benefits of VCT, it is important to determine their awareness and utilization of VCT services, willingness to undergo and pay for VCT so that barriers can be identified and interventions can be planned. Hence this study aimed at assessing the knowledge, attitude and practice of University students towards VCT service in North West Ethiopia.

## Methods

### Study area, study design and study period

This was a cross sectional study conducted among university students of in Ethiopia from February to May, 2010. During the study period there were 10,500 students in three campuses under five faculties.

### Sample size and sampling technique

The campus involved in this study was selected purposefully. During the study period there were 2,900 (917 1st year, 1012 2nd year and 971 3rd year) students enrolled in nine departments in the campus studied. Since there is no similar study in target group in our study area, the sample, the sample size was determined by a single proportion formula using

$$N=\frac{{\left(Z_{\alpha /2}\right)}^{2}X\;P\;\left(1-P\right)}{d^{2}}$$

Where Z—1.96, P—50%, d—0.05, accordingly the sample size will be 384. Since the total population in the campus was 2,900, adjustment was made by the following formula nf = ni/[1 + (ni/N)]; where nf is the sample size calculated by adjustment, ni is the initial sample size calculated by single proportion formula (384), N is the total number of students in the campus (2,900). Based on this formula the sample size for this study was 340.

The sampling technique employed was multistage sampling technique; stratified sampling followed by simple random sampling technique. The list of all students were obtained from the campus’s registrar office and considering students with the same year of study as a homogenous group, they were stratified in to three clusters as year I, year II and year III. Then numbers of study participants for each stratum was allocated proportionally and samples were selected by simple random sampling technique using the list of students as a sample frame. Based on these 95 students from year I, 144 students from year II and 101 students from year III were enrolled.

### Data collection

The study instrument was a self-administered questionnaire which comprised of four parts. Part I related to students’ sociodemographic background, Part II on students’ knowledge regarding VCT, Part III on attitude scale towards VCT and Part IV on students’ practice of VCT.

Knowledge of VCT for HIV was assessed using a five item questionnaire which includes knowledge on the presence of blood test for HIV, counseling service during test, voluntary basis of the test, places where VCT service is provided, importance of VCT in HIV prevention and control. Attitude towards VCT was also assessed using a five item questionnaire which includes attitude: towards screening of HIV, towards necessity of VCT, towards their recommendation of VCT for others and towards eligibility to VCT Practice of VCT was assessed by single question as "Did you used VCT service?” which respond in “Yes” or “No”.

The questionnaire was prepared in English version and translated to the local language (Amharic) and checked for consistency. Before the actual data collection it was pre-tested on twenty students in the other campus of the university and the result was used to improve the phrasing of questions in the questionnaire. Improvement was made on the attitude questions since they looked knowledge questions. Questionnaire validation tests showed that the Alpha Cronbach was 0.85 for knowledge of HIV.

### Scoring

For knowledge about VCT, each question had a response of “Yes” for correct answers or “No” for wrong answers. Scores of all the respondents was sum up and the mean value was calculated. Participants who scored greater than or equal to the mean were considered knowledgeable and the others not knowledgeable. A five-item attitude indicator, responded as either “Yes” or “No”, towards VCT test was used to assess the student’s level of attitude towards VCT. A score serving as a proxy variable was calculated by adding each of the attitudinal scores after giving a value of “1” and “0” for positive and negative responses respectively. Respondents who scored greater than or equal the proxy variable were considered as having positive attitude and those scored less than the proxy variable were considered as having negative attitude . Practice was assessed using one question having “Yes” or “No” response. Those who responded “Yes” were considered as they had had VCT service in the past.

### Data management and analysis

During data collection process, the data were checked for completeness and any incomplete or misfiled questions were sent back for correction. Data were double entered and analyzed using SPSS-16 statistical software. Descriptive statistics were used to give a clear picture of background variables like age, sex and other variables in well structured questionnaire. The frequency distribution of both dependent (knowledge, attitude and practice of VCT) and independent (sociodemographic) variables were worked out. The association between variables was measured and tested using chi square. A P-value < 0.05 was considered to be statistically significant in all cases.

### Ethical consideration

The study was ethically cleared by the research organizing and approving committee of the department of Medical Laboratory Technology, University of Gondar. Written consent, after explanation about the study, was obtained from the study participants. Students thoroughly read the consent from and signed on it. For confidentiality the name of the participants was not typed on the questionnaire. The researchers were from the other campus of the university and had no any relation with the students enrolled in the study.

## Results

### Sociodemographic characteristics of the respondents

Table 1 represents the characteristics of the study participants. A total of 330 (97.3%), subjects completed the questionnaire among whom 218 (66.1%) were males. The mean age of the study participants was 20 (±1.7) years. The majority, 301(91.2%), 170 (51.5%), 250 (75.6%) and 208 (63%) of the respondents were in the age group from 19 to 22 years, Amhara by ethnicity, Orthodox Christians in religion and originate from urban areas respectively (Table 1).

**Table 1:** Sociodemographic characteristics of the study subjects

**Variables**	**Frequency, N (%)**
Sex	
Male	218 (66.1)
Female	112 (33.9)
Age	
15-18	3 (0.91)
19-22	301 (91.2)
> 22	26 (7.88)
Year of study	
1st year	87 (26.4)
2nd year	142 (43)
3rd year	101 (30.6)
Religion	
Orthodox	250 (75.8)
Muslim	45 (13.6)
Protestant	30 (9.1)
Others	5 (1.5)
Residence	
Urban	208 (63.1)
Rural	122 (36.9)
Marital status	
Married	19 (5.76)
Unmarried	311 (94.24)

### Knowledge, attitude and practice of the respondents about VCT

#### Knowledge about VCT

A total of five questions, with “Yes” and “No” response, were included in the questionnaire regarding the knowledge of the study subjects about VCT. The mean of correct answers was computed and those who score a value greater than or equal to the mean were considered knowledgeable. The mean scored value was 4.85. From the respondents 285 (86.3%) scored greater than or equal to the mean and considered knowledgeable. Two hundred and eighty nine (87.6%) of the respondents knew that HIV testing is given with counseling services. The place where VCT service is given is known by 258 (78.2%) of the respondents (Table 2). Mass media is the source of information for about 224 (67.9%) of the respondents. Previous residence of the respondents before joining the university (χ^*2*^ = 26, P-value < 0.001) showed statistically significant association with the knowledge status on VCT service (Table 3).

**Table 2:** Knowledge questions about VCT for HIV and responses given by the respondents

**Questions**	**Responses**	**Frequency (%)**
1. Have you ever heard about VCT?	Yes	310 (93.9)
	No	20 (6.1)
2. Do you know HIV testing is given with counseling?	Yes	289 (87.6)
	No	41 (12.4)
3. Do you know HIV test is conducted voluntarily?	Yes	291 (88.2)
	No	39 (11.8)
4. Do you know the place where VCT is provided?	Yes	258 (78.2)
	No	72 (21.8)
5. Is VCT important for the prevention and control of HIV?	Yes	285 (86.4)
	No	45 (13.6)

**Table 3:** Association between sociodemographic variables with the knowledge of respondents about VCT service

**Variables**	**Knowledgeable (%)**	**Not knowledgeable (%)**	**χ**^**2 **^**(P-value) **
Sex			0.06(0.80)
Male	189 (86.7)	29 (13.3)	
Female	96 (85.7)	16 (14.3)	
Age			
15-18	2 (66.7)	1(33.3)	1.79 (0.41)
19-22	262 (87)	39 (13)	
>22	21 (80.77)	5 (19.23)	
Year of study			
1st year	73 (83.9)	14 (16.1)	1.12 (0.57)
2nd year	122 (85.9)	20 (14.2)	
3rd year	90 (89.1)	11 (10.9)	
Residence			
Urban	195 (93.7)	13 (6.2)	26 (<0.001)
Rural	90 (73.8)	32 (26.2)	
Religion			
Orthodox	217 (86.8)	33 (13.2)	2.99 (0.39)
Muslim	36 (80)	9 (20)	
Protestant	28 (93.3)	2 (6.7)	
Others	4 (80)	1 (20)	

#### Attitude towards VCT

A five-item attitude indicator responded as either “Yes” or “No”, towards VCT test was used to assess the student’s level of attitude towards VCT. A score serving as a proxy variable was calculated by adding each of the attitudinal scores after giving a value of “1” and “0” for positive and negative responses respectively. Accordingly 242 (73.3%) of the respondents had positive attitude towards VCT service. Based on the findings 276 (83.6%) of the respondents felt that VCT is necessary. The most important reasons why VCT is necessary for the respondents include knowing self status and caring for the future, 232 (70.3%), to prevent partners and others from HIV, 179 (54.2%) and to choose partners for the future, 128 (38.8%). Majority, 254 (76.9%), and 244 (73.9%), of the respondents would recommend VCT to their partners and are interested to get VCT service for themselves (Table 4). The educational level of respondents and previous residence of respondents before joining the university had a statistically significant association with the attitude towards VCT service at *χ*^*2*^ (P-value) of 15.8 (<0.001 and 12 (<0.001) respectively. The knowledge of respondents about VCT has also showed an association with attitude towards VCT (χ^2^ = 30.026, P-value < 0.001) (Table 5).

**Table 4:** Responses given by the respondents to attitude questions towards VCT for HIV

**Questions**	**Responses**	**Frequency (%)**
1. Do you think that VCT is necessary?	Yes	276 (83.6)
	No	54 (16.4)
2. Do you recommend VCT for others?	Yes	254 (76.9)
	No	76 (23.03)
3. Are you interested to take VCT whether you have it before or not?	Yes	244 (73.9)
	No	86 (26.1)
4. Does that VCT site affects your interest to take VCT?	Yes	255 (72.3)
	No	75 (22.7)
5. Do you have any preference of individuals in VCT sites to take the service?	Yes	200 (60.6)
	No	130 (39.4)

**Table 5:** Association between sociodemographic variables and knowledge on VCT with the attitude of respondents towards VCT service

**Variables**	**Positive attitude**	**Negative attitude**	**χ**^**2 **^**(P-value)**
Sex			
Male	167 (76.6)	51 (23.4)	1.92 (0.26)
Female	75 (66.9)	37 (33)	
Age			
15-18	2 (66.6)	1 (33.3)	0.28 (0.88)
19-22	220 (73.1)	81 (26.9)	
> 22	20 (76.9)	6 (23.1)	
Year of study			
1st year	51 (58.6)	36 (41.4)	15.8 (<0.001)
2nd year	106 (74.6)	36 (25.3)	
3rd year	85 (84.1)	16 (15.8)	
Residence			
Urban	166 (79.8)	42 (20,2)	12.06 (<0.001)
Rural	76 (62.3)	46 (37.7)	
Religion			
Orthodox	188 (75.2)	62 (24.8)	6.81(0.078)
Muslim	26 (57.8)	19 (42.2)	
Protestant	24 (80)	6 (20)	
Others	4 (80)	1 (20)	
Knowledge on VCT			
Knowledgeable	226	59	30.026 (<0.001)
Not knowledgeable	16	29	

#### Practice of VCT for HIV

Regarding the practices on VCT, 204 (61.8%) had had VCT in the past. Among those who had used VCT 151(74%) were satisfied with the counseling service they got. Fear of positive results, fear of stigma and discrimination, partner and self trust and partner refusal were the reasons mentioned for not attending VCT services by 106 (84.1%), 74 (58.7%), 18 (14.8%) and 6 (4.8%) of the respondents respectively (Table 6). Among the sociodemographic variables sex (*χ*^*2*^ = 7.2, P-value < 0.05), previous residence before joining the university (*χ*^*2*^ = 9.9, P-value < 0.01) and religion (*χ*^*2*^ = 28.9, P-value < 0.0001) had a statistically significant association with the habit of using VCT service. Additionally knowledge about VCT (*χ*^*2*^ = 15.23, P-value < 0.001) and attitude towards VCT (*χ*^*2*^ = 153.8, P-value < 0.001) showed a statistically significant association with the practice of VCT for HIV (Table 7).

**Table 6:** Respondents practice of VCT for HIV

**Questions**	**Responses**	**Frequency (%)**
1. Have you ever had VCT in the past?	Yes	204 (61.8)
	No	126 (38.2)
2. What were your reasons for not having VCT?	Fear of stigma and discrimination	74 (58.7)
	Fear of positive results	106 (84.1)
	Partner and self trust	18 (14.8)
	Partner refusal	6 (4.8)
3. Where did you take VCT service?	Hospital	102 (50)
	Health center	83 (40.7)
	NGO clinic	10 (4.9)
	Private clinic	9 (4.4)

**Table 7:** Association between sociodemographic variables, knowledge about VCT and attitude towards VCT with practice of VCT service for HIV

**Variables**	**Practice VCT**	**Do not practice VCT**	**χ**^**2 **^**(P-value)**
Sex			
Male	146 (66.9)	72 (33)	7.23 (<0.01)
Female	58 (51.8)	54 (48.2)	
Age			
15-18	1 (33.3)	2 (66.6)	1.85 (0.39)
19-22	189 (62.8)	112 (37.2)	
>22	14 (53.8)	12 (46.2)	
Year of study			
1st year	54 (62.1)	33 (37.9)	4.2 (0.12)
2nd year	80 (56.3)	62 (43.7)	
3rd year	70 (69.3)	31 (30.7)	
Residence			
Urban	142 (68.3)	66 (31.7)	9.92 (0.001)
Rural	62 (50.8)	60 (49.2)	
Religion			
Orthodox	172 (68.8)	78 (31.2)	28.31 (<0.001)
Muslim	13 (28.9)	32 (71.1)	
Protestant	15 (50)	15 (50)	
Others	4 (80)	1 (20)	
Knowledge on VCT			
Knowledgeable	188	97	15.23 (<0.001)
Not knowledgeable	16	29	
Attitude towards VCT			
Positive attitude	198	44	153.8 (<0.001)
Negative attitude	6	82	

## Discussion

From the finding of this study the overall knowledge of respondents about VCT for HIV was high. A comparable report was found in Ethiopia from high school students [19]. Though the degree of awareness varies, reports from Nigeria and Tanzania indicated a good level of knowledge about VCT among tertiary school students [7,8,20]. But a community based research from China indicated a significant level of lack of knowledge about HIV and VCT on HIV [21]. This might be due to the difference in the sociodemographic characteristics, especially the educational level, of the study participants. Assessment on the impact of sociodemographic variables on the knowledge of VCT showed that previous resident area before joining the university has significant association with the level of knowledge. The difference in the level of knowledge between students originated from urban and rural might be due to the accessibility of information to the rural set up is minimal and this urges the need of further activities on awareness creation about VCT in the rural areas of the country.

The participants attitude towards VCT was very high and appreciable which is very important and needed in the prevention and control of HIV. Majority believed that VCT is necessary for different reasons including knowing self status and caring for the future, to prevent partners and others from HIV and to choose partners for the future. The level of educational and previous residence of students was sociodemographic variables that showed a significant association with attitude of students towards VCT. Students from rural areas lack good level of information about VCT and may be associated with decreased level of attitude, though not statistically significant. The association of level of education may rise from the increase in knowledge of students and hence increased attitude as the stay in the university is elongated. The findings in this case go in line with a study from Addis Ababa University [22]. The current report is also similar to a study from Northern Nigeria where majority of the adults were volunteer to get VCT service and would recommend VCT to their friends [23].

The results from this study also indicated a relatively good attendance of VCT which is much higher than a study conducted in Nigeria, Tanzania and Uganda [8,24]. The difference may be attributable to one’s health beliefs and health seeking behavior, cultural, beliefs, social networks, income, perceived health status and severity of disease. According to this study the main reason for those who had never had VCT in the past was fear of positive result and stigma and discrimination following the result. Though there is a difference in VCT attendance main reasons for not visiting VCT centers is similar across different study areas including community based surveys [14,19,20,25,26]. This indicated the need of more work in awareness creation associated with stigma and discrimination and the possibility to live longer with the virus as far as HIV positive individuals lead their lives as per physicians and counselors recommendations. According to the current study sex, previous residence before joining the university and religion showed statistically significant association with level of VCT uptake. Moreover knowledge about HIV, knowledge about VCT and attitude towards VCT showed association with practice on VCT for HIV which indicates the relation of one with the other. Hence, working on the knowledge and attitude change will facilitate the uptake of VCT service.

### Limitation of the study

The findings of our study must be interpreted in the context of the limitations encountered. This was a questionnaire-based cross-sectional study in which we relied completely on information provided by the respondents, which may lead to bias and misunderstanding of questions. Our study was a single institutional survey and the results may not be generalized to the majority of tertiary level students. Perceived risk of HIV was not assessed which will strengthen the result of this study. The use of mean to classify as knowledgeable and not knowledgeable may contribute to the bias in this study. Nevertheless, it provides useful information for planning a multicenter study.

## Conclusion

The current study sought to explore the knowledge, attitude and practice on VCT of tertiary school students in one of the oldest universities in the country. Since VCT is an important tool for HIV prevention and control knowing the level of uptake of the service by this high risk group is vital in future activities to be conducted. From this study it can be concluded that there is a need to work on awareness creation on stigma and discrimination since they were among the determining factors for the uptake of VCT. More over building the knowledge of people in rural areas about VCT should be an area of focus to work on. Expansion of facilities that provide VCT service and training of counselors are also important activities that must be done to improve utilization of the service.

## Competing interests

The authors do not have any competing interests to declare.

## Authors’ contributions

ZA conceived the study, undertook statistical analysis and drafted the manuscript. AY, YS, AA, WB, BM and BT initiated the study and made major contributions to the study design and statistical analysis. All authors contributed to the writing of the manuscript and approved the submitted version of the manuscript.

## Pre-publication history

The pre-publication history for this paper can be accessed here:

http://www.biomedcentral.com/1471-2458/13/714/prepub

## References

[B1] Kallings LO The first postmodern pandemic: 25 years of HIV/AIDS J Intern Med 2008 263 218 243 10.1111/j.1365-2796.2007.01910.x 18205765

[B2] WHO Global HIV/AIDS response: epidemic update and health sector progress towards universal access Progress report 2011 http://www.unaids.org/en/media/unaids/contentassets/documents/unaidspublication/2011/20111130_ua_report_en.pdf

[B3] UNAIDS How to get zero faster, smarter, better, World AIDS day report Joint United Nations program on HIV/AIDS 2011 http://www.unaids.org/en/media/unaids/contentassets/documents/unaidspublication/2011/jc2216_worldaidsday_report_2011_en.pdf

[B4] Imanguli N Youth and the global HIV/AIDS pandemic 2008 Advocates for youth http://www.advocatesforyouth.org/storage/advfy/documents/fsglobalhiv.pdf

[B5] Federal Ministry of Health, Ethiopia Strategic plan for intensifying multisectoral HIV and AIDS response in Ethiopia II (SPM II) 2009–2014 2009 Federal HIV/AIDS Prevention and Control Office

[B6] Central Statistical Agency [Ethiopia] and ICF International Ethiopia demographic and health survey 2011 2012 Addis Ababa, Ethiopia and Calverton, Maryland, USA

[B7] Charles PM Kweka JE Mahande MA Barongo RL Shekalaghe S Nkya MH Lowassa A Mahande JM Evaluation of uptake and attitude to voluntary counseling and testing among health care professional students in Kilimanjaro region, Tanzania BMC Public Health 2009 9 128 10.1186/1471-2458-9-128 19426514 PMC2684744

[B8] Uzochukwua B Ugurub N Ezeokea U Onwujekweb O Sibeuduc T Voluntary counseling and testing (VCT) for HIV/AIDS: A study of the knowledge, awareness and willingness to pay for VCT among students in tertiary institutions in Enugu State Nigeria Health Policy 2011 90 277 284 10.1016/j.healthpol.2010.11.007 21130516

[B9] UNAIDS Voluntary Counseling and Testing (VCT): Technical Update 2000

[B10] Admassu M Fitaw Y Factors affecting acceptance of VCT among different professional and community groups in North and South Gondar administrative zones, North West Ethiopia Ethiop J Health Dev 2006 20 24 31

[B11] Federal Ministry of Health, Ethiopia Guidelines for HIV counseling and testing in Ethiopia 2007 Federal HIV/AIDS Prevention and Control Office

[B12] Li X Lin C Gao Z Stanton B Fang X Yin Q Wu Y HIV/AIDS knowledge and the implications for health promotion programs among Chinese college students: geographic, gender and age differences Health Promot Int 2004 19 345 356 10.1093/heapro/dah308 15306619

[B13] Shankar PR Subish P Paudel R Alam K Perception and knowledge about HIV/AIDS among students in a medical college in Western Nepal SAARC J Tuber Lung Dis HIV/AIDS 2009 2 11 16

[B14] Yahaya LA Jimoh AAG Balogun OR Factors hindering acceptance of HIV/AIDS Voluntary Counseling and Testing (VCT) among youth in Kwara State, Nigeria Afr J Reprod Health 2010 14 159 164 21495608

[B15] Alemu S Abseno N Degu G Wondmikun Y Amsalu S Knowledge and attitude towards voluntary counseling and testing for HIV: a community based study in Northwest Ethiopia Ethiop J Health Dev 2004 18 82 89

[B16] Duncan C Miller DM Borskey EJ Barriers to safer sex practices among African American college students J Natl Med Assoc 2002 94 944 951 12442997 PMC2594191

[B17] Prince A Bernard AL Sexual behaviors and safer sex practices of college students at a commuter campus J Am Coll Health 1998 47 1 11 21 10.1080/07448489809595614 9693475

[B18] Lewis JE Malow RM Ireland SJ HIV/AIDS risk in heterosexual college students-a review of a decade of literature J Am Coll Health 1997 45 4 147 158 10.1080/07448481.1997.9936875 9019001

[B19] Abebe A Mitikie G Perception of high school students towards voluntary HIV counseling and testing, using health belief model in Butajira, SNNPR Ethiop J health Dev 2009 23 148 153

[B20] Daniyam AC Agaba AP Agaba IE Acceptability of voluntary counseling and testing among medical students in Jos, Nigeria J Infect Dev Ctries 2010 4 357 361 20601786

[B21] Maa W Detelsb R Fengb Y Wua Z Shenc L Lic Y Lid Z Chene F Wange A Liud T Acceptance of and barriers to voluntary HIV counselling and testing among adults in Guizhou province, China AIDS 2007 21 Suppl 8 129 135 10.1097/01.aids.0000304708.64294.3f PMC2903547 18172381

[B22] Regassa N Kedir S Attitudes and practices on HIV preventions among students of higher education institutions in Ethiopia: The case of Addis Ababa University Educ Res 2011 2 828 840 22066302

[B23] Lliyasu Z Abubakar SI Kabir M Aliyu HM Knowledge of HIV/AIDS and attitude towards Voluntary Counseling and Testing among adults J Nat Med Assoc 2006 98 1912 1922 PMC2569677 17225834

[B24] Vermeer W Bos ERA Mbwamboc J Kaaya S Schaalma PH Social and cognitive variables predicting voluntary HIV counseling and testing among Tanzanian medical students Patient Educ Couns 2009 75 135 140 10.1016/j.pec.2008.08.022 18951747

[B25] Peltzer K Mpofu E Baguma P Lawal B Attitudes towards HIV-antibody testing among university students in four African countries Int J Adv Couns 2002 24 193 203 10.1023/A:1022991522264

[B26] Meiberg AE Bos ERA Onya EH Schaalma PH Fear of stigmatization as barrier to voluntary HIV counselling and testing in South Africa East Afr J Public Health 2008 5 49 54 19024410

